# Upper limb intelligent feedback robot training significantly activates the cerebral cortex and promotes the functional connectivity of the cerebral cortex in patients with stroke: A functional near-infrared spectroscopy study

**DOI:** 10.3389/fneur.2023.1042254

**Published:** 2023-02-06

**Authors:** Hao Li, Xuefeng Fu, Lijun Lu, Hua Guo, Wen Yang, Kaifeng Guo, Zhen Huang

**Affiliations:** ^1^Guangzhou Panyu Central Hospital, Guangzhou, China; ^2^Guangzhou University of Chinese Medicine, Guangzhou, China

**Keywords:** upper limb intelligent feedback robot, functional near-infrared spectroscopy, stroke, shoulder joint training, cerebral cortex activation, functional connectivity

## Abstract

**Background:**

Upper limb intelligence robots are widely used to improve the upper limb function of patients with stroke, but the treatment mechanism is still not clear. In this study, functional near-infrared spectroscopy (fNIRS) was used to evaluate the concentration changes of oxygenated hemoglobin (oxy-Hb) and deoxyhemoglobin (deoxy-Hb) in different brain regions and functional connectivity (FC) of the cerebral cortex in patients with stroke.

**Method:**

Twenty post-stroke patients with upper limb dysfunction were included in the study. They all received three different types of shoulder joint training, namely, active intelligent feedback robot training (ACT), upper limb suspension training (SUS), and passive intelligent feedback robot training (PAS). During the training, activation of the cerebral cortex was detected by fNIRS to obtain the concentration changes of hemoglobin and FC of the cerebral cortex. The fNIRS signals were recorded over eight ROIs: bilateral prefrontal cortices (PFC), bilateral primary motor cortices (M1), bilateral primary somatosensory cortices (S1), and bilateral premotor and supplementary motor cortices (PM). For easy comparison, we defined the right hemisphere as the ipsilesional hemisphere and flipped the lesional right hemisphere in the Nirspark.

**Result:**

Compared with the other two groups, stronger cerebral cortex activation was observed during ACT. One-way repeated measures ANOVA revealed significant differences in mean oxy-Hb changes among conditions in the four ROIs: contralesional PFC [F_(2, 48)_ = 6,798, *p* < 0.01], ipsilesional M1 [F_(2, 48)_ = 6.733, *p* < 0.01], ipsilesional S1 [F_(2, 48)_ = 4,392, *p* < 0.05], and ipsilesional PM [F_(2, 48)_ = 3.658, *p* < 0.05]. Oxy-Hb responses in the contralesional PFC region were stronger during ACT than during SUS (*p* < 0.01) and PAS (*p* < 0.05). Cortical activation in the ipsilesional M1 was significantly greater during ACT than during SUS (*p* < 0.01) and PAS (*p* < 0.05). Oxy-Hb responses in the ipsilesional S1 (*p* < 0.05) and ipsilesional PM (*p* < 0.05) were significantly higher during ACT than during PAS, and there is no significant difference in mean deoxy-Hb changes among conditions. Compared with SUS, the FC increased during ACT, which was characterized by the enhanced function of the ipsilesional cortex (*p* < 0.05), and there was no significant difference in FC between the ACT and PAS.

**Conclusion:**

The study found that cortical activation during ACT was higher in the contralesional PFC, and ipsilesional M1 than during SUS, and showed tighter cortical FC between the cortices. The activation of the cerebral cortex of ACT was significantly higher than that of PAS, but there was no significant difference in FC. Our research helps to understand the difference in cerebral cortex activation between upper limb intelligent feedback robot rehabilitation and other rehabilitation training and provides an objective basis for the further application of upper limb intelligent feedback robots in the field of stroke rehabilitation.

## Introduction

Stroke is a major chronic noncommunicable disease that seriously endangers the health of Chinese people, and it has five characteristics: high morbidity, high disability rate, high mortality rate, high recurrence rate, and high economic burden. In 2018, the mortality rate of cerebrovascular diseases in China was 149.49 per 100,000, and the death toll was 1.57 million (1). As one of the most common complications of stroke, upper limb motor dysfunction hinders patients from returning to family and society to a great extent and has a serious impact on their physical and mental health. More than two-thirds of people have decreased upper limb function when they are discharged from the hospital after stroke (2). About half of the affected upper limbs are still dysfunctional after 6 months (3).

In recent years, upper limb rehabilitation robots have been widely used in the recovery of patients with stroke, and their curative effect has been confirmed by a large number of studies (4). In the process of clinical training, the upper limb rehabilitation robot mainly provides four sports training modes: resistance movement, active movement, auxiliary movement, and passive movement (5). A reasonable training mode can be chosen according to the degree of active participation of the patients. The current research found that upper limb rehabilitation robots can mainly improve upper limb muscle strength, range of motion, and motor function but have less improvement in muscle tension and activities of daily living (6). Many previous studies have shown that one of the advantages of robotic equipment may be due to the increase in training motivation, the opportunity for independent movement, and the increase in the number of repetitions during arm training (7). Similarly, this intensive, frequent, and repetitive treatment model is also in line with the principle of motor learning so that it can produce a better effect than conventional rehabilitation. At present, some teams have used electroencephalography (EEG) (8), transcranial magnetic stimulation (TMS) (9), functional magnetic resonance imaging (fMRI) (10), and other noninvasive neuroimaging to study its therapeutic mechanism. The aforementioned techniques have various shortcomings, such as high cost, limited experimental sites, and unsuitable for continuous monitoring, which hinder the in-depth study of brain function response in the process of upper limb functional recovery.

Stroke leads to damage to the cerebral cortex, but the brain has neuroplasticity, which is including the reorganization of the brain structure and function, and increasing the movement of the affected limb helps patients overcome the previously learned non-use for the affected limb and promotes use-dependent plastic brain reorganization, so it can accelerate recovery of the nerve (11). Compared with bilateral training, the coupling effect between the ipsilesional and contralesional motor cortices was significantly enhanced in unilateral training. In addition, the local efficiency of the ipsilesional motor cortex, ipsilesional prefrontal cortex (PFC), and contralesional PFC and the hemispheric autonomy index of ipsilesional PFC were significantly increased (12). In cycling exercises with visual speed feedback, there is more concentrated activation of the premotor cortex and better motor performance than those without visual feedback (13). Even without active movement, the left PFC, bilateral premotor cortex, and supplementary motor area were significantly activated when healthy adult subjects imaged normal standing balance tasks and dynamic standing balance tasks (14). It is proved that the higher the subjective participation of patients is, the stronger the brain activation is. With the remodeling of the brain, the abnormal activation pattern will also be improved, which is characterized by a decrease in overall activation and an improvement in the lateral index (LI), and the affected side of the brain will slowly restore the innervation of the limbs (15).

Functional near-infrared spectroscopy (fNIRS) is a noninvasive imaging technique based on optical principle, which makes use of the strong penetration of near-infrared light (650~950 nm) to biological tissue and can reach the cerebral cortex of intracranial 2~3 cm (16). The dominant and physiologically dependent absorption chromophore in the biological tissue is hemoglobin, which is in the form of oxygenated hemoglobin (oxy-Hb) and deoxyhemoglobin (deoxy-Hb), respectively, and they have distinguishable light absorption properties in this spectral window. According to the correlation between light decay and chromophore concentration changes in tissue, fNIRS can quantitatively analyze the concentration changes of oxy-Hb and deoxy-Hb in the brain tissue (17). Based on the neuro-vascular coupling mechanism, the functional activity of the brain is accompanied by the activation of the cerebral cortex, which will cause changes in regional blood oxygen metabolic rate and cerebral hemodynamics, and the increased rate of regional cerebral blood flow in the activated region of the brain is much higher than that of the local oxygen consumption rate, which is finally characterized by the increase of oxy-Hb concentration and the decrease of deoxy-Hb concentration in the activated region (18). Therefore, fNIRS can indirectly monitor the functional activity of the cerebral cortex through the changes in oxy-Hb concentration and deoxy-Hb concentration.

The fNIRS has the advantages of a relatively low price, high time resolution, suitable for more places, and less noise interference. At present, fNIRS is mainly used to observe the changes in cortical activation in subjects under different conditions and after treatment, which strengthens the study of cerebral activity and reveals the principle of cortical remodeling (19). For example, the fNIRS showed that the asymmetry of sensorimotor cortex activation was significantly improved after 2 months of in-hospital rehabilitation, and the ipsilesional premotor cortex activation increased, suggesting that the motor recovery after stroke may be related to the improvement of sensorimotor cortex (SMC) activation asymmetry and the enhancement of ipsilesional premotor cortex activation (15). In addition, it can also be used to observe the functional connectivity (FC) between the cerebral cortex of the subjects during the task (20).

In conclusion, upper limb intelligent feedback robots and fNIRS are both used in basic research and clinical treatment, but there is little research on the use of fNIRS to compare the changes in cerebral cortex activation between upper limb intelligent feedback robot rehabilitation and other rehabilitation methods. The purpose of this study is to compare the activation of the cerebral cortex and cerebral cortex network in different shoulder training processes by fNIRS and to explore the mechanism of upper limb intelligent feedback robot training. We hypothesized that the changes in cerebral cortex activation and FC of active intelligent feedback robot training were significantly stronger than upper limb suspension training and passive intelligent feedback robot training.

## Materials and methods

### Participants

In total, 20 patients with stroke (nine women and 11 men, age range: 59.55 ± 10.28 years) were enrolled in this study.

The inclusion criteria for these patients were as follows: (a) meeting the stroke diagnostic criteria of Chinese guidelines for the prevention and treatment of cerebrovascular diseases; cerebral infarction or cerebral hemorrhage confirmed by CT or MRI; (b) patients with the first onset, a course of the disease within 2 years, aged 30–70 years old, regardless of gender; (c) limitation of upper limb function, Brunnstrom stage ≥ II, muscle strength ≥ III, sitting balance ≥ II; (d) right-handed individuals as identified in the Edinburgh Handedness Inventory; and (e) no serious cognitive problems, and Mini-Mental State Examination (MMSE) score of ≥21 according to education level. The exclusion criteria were as follows: (a) medications that reduce seizure thresholds or psychotropic medications; (b) patients with shoulder pain, dislocation, or other underlying diseases, such as a fracture; (c) metal implants in skulls, missing skulls, or other patients who cannot be tested for fNIRS; and (d) a history of mental illness or taking any antipsychotic drugs that are not suitable for this study. The exclusion and drop-out criteria were as follows: (a) patients who did not cooperate with the test or examination and had poor compliance and (b) those who withdrew from the trial voluntarily.

A total of 20 hemiplegic patients were recruited, and 16 patients completed this study. The stroke duration ranged from 1 to 24 months (median time = 5.1 months). Notably, 14 patients had stroke in the right hemisphere (70%), and six patients in the left hemisphere (30%) (Table 1). The experiment was conducted following the ethical principles of the Declaration of Helsinki (21). All participants provided written informed consent before data collection. The study was approved by the Ethics Committee of Guangzhou Panyu Central Hospital (No.: PYRC-2021-008).

**Table 1:** Demographics and clinical properties of the participants.

**Number**	**Age**	**Gender**	**Hemisphere lesion**	**Type of lesion**	**Days after stroke(m)**	**MMSE**
1	58	F	R pons	CI	1	24
2	62	F	R radial crown	CI	2	23
3	51	F	R basal ganglia	ICH	8	25
4	79	M	R hemisphere	ICH	9	22
5	47	M	R hemisphere	CI	4	26
6	53	F	R thalamus	ICH	8	25
7	66	M	R basal ganglia	CI	18	25
8	66	M	L thalamus	ICH	1	23
9	63	F	L thalamus	ICH	2	24
10	55	F	L pons	CI	1	24
11	42	F	L basal ganglia	ICH	1	26
12	67	M	L pons	CI	1	23
13	58	M	R thalamus	ICH	1	24
14	65	M	R basal ganglia and radial crown	CI	5	24
15	61	F	R radial crown	CI	1	25
16	37	M	R frontal lobe and temporal lobe	CI	1	27
17	59	M	L basal ganglia	ICH	24	24
18	59	M	R MCAO	CI	9	25
19	76	F	R parietal lobe	CI	3	23
20	67	M	R brainstem	CI	2	25

M, male; F, female; R, right; L, left; MCAO, middle cerebral artery occlusion; ICH, Intracerebral Hemorrhage; CI, Cerebral Infraction; MMSE, Mini-mental State Examination.

### Apparatus

**Figure 1:**
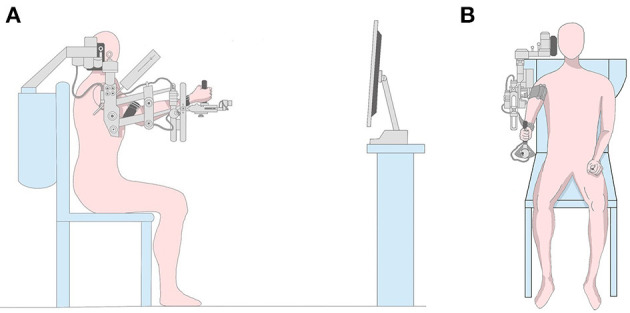
Experimental setup. **(A)** Experimental setup from a sagittal view. The patient sat in a chair and two meters in front was a display screen. **(B)** Experimental setup from a coronal view. All parts of the affected limb were fixed and shoulder adductor abduction movement was performed in the task state.

The intelligent feedback robot training system (Yikang Co., China) is composed of an intelligent feedback robot and a multimedia display screen. The intelligent feedback robot includes the shoulder, the elbow, and the wrist, which can support all the weight of the patient's upper limb, and the joint angle can be adjusted for different patients. There is a chair beside the robot that allows patients to train their upper limbs in the sitting position. Two meters directly in front of the robot is a multimedia display screen, which can display dynamic game content, reflect the robot's movements, and play audio (Figure 1). During the training, the patient adduced or abducted the affected shoulder joint according to the multimedia game and then received different visual and auditory feedback at the same time.

### Task conditions

We detected cerebral cortical activation during the three training sessions. All three types of training were carried out on the upper limb intelligent feedback robot system, namely, active intelligent feedback robot training (ACT), upper limb suspension training (SUS), and passive intelligent feedback robot training (PAS). Before training, the patient was fixed on the seat, and a therapist turned on the robot system. Then, the robot arm was fixed, and the height of the upper arm was adjusted with nuts. Finally, the components at the shoulder, elbow, and wrist were worn in turn. During ACT, patients received feedback from the game and actively completed the training. During PAS, patients received game feedback and performed motor imagination but completed the training passively. During SUS, patients actively completed the training without game feedback. Both ACT and PAS played the game, namely, “catching the coins,” which required patients to adduct and abduct their shoulder joints to collect falling coins. The experimental design comprised two periods, namely, the resting state (RS) and task state (TS). The display screen of TS had sound and image playing, while RS had no sound and image, and stood still. During SUS, patients actively adducted and abducted shoulder joints without feedback in the TS and rested in the RS. The motion velocities of the three mission states were consistent.

### Task procedure

**Figure 2:**
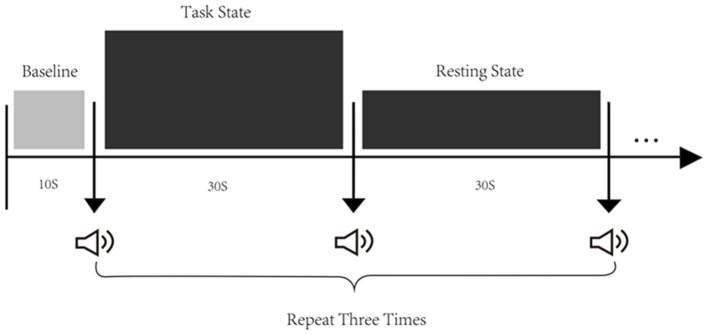
Experimental procedure. There are three states of fNIRS testing, namely, the baseline, the task state (TS), and the resting state (RS). The first 10 s before the test were taken as the baseline. Then, start three tests, each including a 30-s task and a 30-s resting.

The fNIRS testing consisted of three consecutive trials in a block paradigm, carried out as follows: Baseline—Task state—Resting state (Figure 2). Each task trial commenced with a baseline period during which patients were required to relax the whole body for 10 s, and then, baseline hemodynamic responses were recorded. Before the test, patients were instructed to sit quietly on the chair, relax with their eyes closed, and not to carry out motor imagination during the RS. Patients could only perform shoulder joint activities, and other physical activities were prohibited to prevent interference with the blood oxygen data during the TS. We ensured that patients fully understood and consented to the training. In addition, the training room was bright, tidy, and quiet, with a temperature of 26–27°C and a humidity of 40–60%. During the training, we collected the first 10 s of data as the baseline and then started the task. The patients performed shoulder joint adduction and abduction for 30 s and then rested for 30 s to return to the baseline, which was a cycle. The training included three cycles, and it took approximately 190 s in total.

### fNIRS data acquisition and analysis

**Figure 3:**
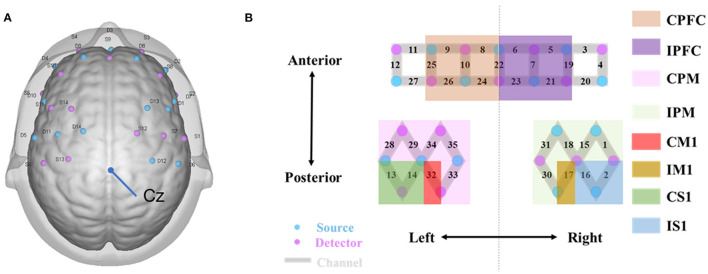
fNIRS data acquisition. **(A)** fNIRS optode layout design. Blue and purple-filled circles represent light sources and detectors, respectively. Gray rectangles represent long separation channels. The midline central point (Cz) is located at the middle point of optode S13 and optode D12. **(B)** Regions of interest and the channel setting. The probes were located over bilateral PFC (CPFC and IPFC); PM (CPM and IPM); bilateral M1 (CM1 and IM1); bilateral S1 (CS1 and IS1). C, contralesional; I, ipsilesional; PFC, prefrontal cortex; PM, premotor area and supplementary motor cortex; M1, primary motor cortex; S1, primary somatosensory cortex.

A multichannel fNIRS system (NirSmart-6000A, Danyang Huichuang Medical Equipment Co., Ltd., Jiangsu, China) with the wavelength of 730 nm and 850 nm was used to detect the changes in concentration of oxy-Hb and deoxy-Hb during the TS and RS. Data were sampled at a frequency of 10 Hz. The distance between the detector and the source was 30 mm to ensure propagation to the gray matter beneath the optodes. In this study, the fNIRS channels were defined as the midpoint of the corresponding light source-detector pairs. A total of 35 channels were built by 14 light sources and 14 detectors for fNIRS measurement. These channels were symmetrically distributed in the left and right hemispheres of the participants (17 channels on each side, and the S9-D3 channel was evenly distributed in the left and right hemispheres) and positioned based on the 10/20 international electrode placement system (Figure 3A). The location of these channels covered the ipsilesional and contralesional prefrontal cortices (IPFC/CPFC), ipsilesional and contralesional primary motor cortices (IM1/CM1), ipsilesional and contralesional primary somatosensory cortices (IS1/CS1), and ipsilesional and contralesional premotor and supplementary motor cortices (IPM/CPM) (Figure 3B). The midline central point (Cz) was located at the midpoint of optode S13 and optode D12. The channel sets for regions of interest (ROI) were selected based on Brodmann areas (BA) and anatomical locations of cortical areas for each participant (Table 2). The acquired coordinates were then transformed into the Montreal Neurological Institute (MNI) coordinates and further projected to the MNI standard brain template using a spatial registration approach in NirSpace (Danyang Huichuang Medical Equipment Co., Ltd., China).

**Table 2:** Anatomically labeled fNIRS channel locations using Brodmann areas.

**Channels**	**Brodmann areas (BA)**	**Regions of interest (ROI)**	**Abbreviation**
2, 16	BA 1, 2, 3	Ipsilesional primary somatosensory cortex	IS1
13, 14	BA 1, 2, 3	Contralesional primary somatosensory cortex	CS1
17	BA 4	Ipsilesional primary motor cortex	IM1
32	BA 4	Contralesional primary motor cortex	CM1
1, 15, 18, 30, 31	BA 6	Ipsilesional premotor and supplementary motor cortex	IPM
28, 29, 33, 34, 35	BA 6	Contralesional premotor and supplementary motor cortex	CPM
5, 6, 7, 19, 21, 22, 23	BA 10	Ipsilesional prefrontal cortex	IPFC
8, 9, 10, 22, 24, 25, 26	BA 10	Contralesional prefrontal cortex	CPFC

fNIRS, functional near-infrared spectroscopy; ROIs, regions of interest; BA, Brodmann Areas; I, ipsilesional; C, contralesional; S1, primary somatosensory cortex; M1, primary motor cortex; PM, premotor area and supplementary motor cortex; PFC, prefrontal cortex.

The calibration function of the instrument and corresponding template was used to ascertain the channels to fill exactly in correspondence with the 10/20 electrode positions. Each optode was attached to the surface of the skull using a custom-made hard plastic cap and covered with a black cloth to prevent penetration of ambient light. The hair was carefully swept away to ensure that the light tube touched the participant's skin closely, thereby maximizing the efficiency of light coupling to the tissue.

In this study, oxy-Hb was used as a marker for hemodynamic changes activated by the local cortex, as previous studies have shown that oxy-Hb is more sensitive to changes in cerebral regional blood flow signal than changes in deoxy-Hb concentration (22, 23). In addition, oxy-Hb is considered to be particularly sensitive to locomotion-related changes (24, 25).

The NirSpark software (HuiChuang, China) package was used to preprocess fNIRS signals, which has been used in previous experiments (26), and the steps were performed as follows. First, an expert performed a preliminary inspection of the raw data, marking and rejecting poor-quality signals. Second, we used a spline interpolation algorithm for the resulting signals to amend motion artifacts by channels. Spline interpolation is a commonly used correction method. The advantage of it is that only corrects the pre-localized artifacts. Motion artifacts were manifested as impulse or cliff-type jumps caused by the relative sliding of the scalp and probes (27). Subsequently, further analysis of the oxy-Hb data and deoxy-Hb of channels covering functionally involved areas, namely, the eight ROIs, was performed, and during the preprocessing, the raw data were band-pass filtered between 0.01 and 0.2 Hz to remove physiological noise (e.g., respiration, cardiac activity, and low-frequency signal drift). Then, the modified Beer–Lambert law was used to calculate the relative hemoglobin concentration changes in oxy-Hb and deoxy-Hb. The hemodynamic response function (HRF) initial time was set to −10 s, and the end time was set to 180 s with “−10–0 s” as the reserved baseline state and “0–60 s” as the time for a single block paradigm. The oxyhemoglobin concentrations for each block paradigm were superimposed and averaged to generate a block average result. For each pre-treated experimental dataset, a generalized linear model (GLM) was used to analyze the oxy-Hb and deoxy-Hb time series data. Finally, we extracted a 3-min stable hemoglobin time series for each participant.

The functional connectivity matrix was computed in NirSpark and calculated by conducting Pearson's correlation analysis between the time series for each pair of channels. For each participant, we generated a 35 × 35 correlation matrix. We used the z matrix for the next calculation step due to its normality characteristics and normalized the correlation coefficients (r) to the z-value using Fisher's r-to-z transformation method. A predetermined sparsity was used to express the actual number of connections divided by the maximum possible number of connections in the network, and then, the correlation matrix was thresholded into a binary matrix describing the topology of the functional network. As in previous studies (28, 29), the sparsity of the brain network should not be too high or too low, and the range of 0.15–0.25 is more appropriate (30), so this study chose a sparsity of 0.2 to construct the brain network. Then, according to BA, our measurement channels were grouped into four regions: PFC, PM, S1, and M1. After considering the brain hemisphere factor, we obtained eight regions in total. Finally, to evaluate the functional connections between and within networks, the z values of the FC matrix were averaged separately, resulting in an 8^*^8 functional connectivity matrix. For easy comparison, we defined the right hemisphere as the ipsilesional hemisphere and flipped the lesional right hemisphere in the Nirspark.

### Statistical analysis

Measurement data were expressed by mean and standard deviation. Data normality was tested *via* the Shapiro–Wilk test. For the analysis of cortical activation, the changes in the oxy-Hb and deoxy-Hb among three conditions were used. A one-way repeated-measures analysis of variance (ANOVA) test was used to examine the effect of condition indices of cortical activation (mean oxy-Hb and deoxy-Hb changes and average effect sizes of hemodynamic response) for each ROI. Bonferroni *post hoc* tests were applied for multiple comparisons.

Pearson's correlation coefficient described the FC. We performed the paired *t*-test to compare the FC between ACT and SUS or ACT and PAS. The statistical results were corrected for multiple comparisons across channels by the false discovery rate (FDR). The statistically significant level was 0.05 in this study.

## Results

A total of 20 patients were recruited and received three different types of training. Two patients were unable to complete three types of training because of fatigue, and the other two patients were excluded because of serious exercise compensation interference with the measurement results. Finally, 16 patients completed three types of training and fNIRS measurements in the process. No adverse effects were reported.

### Cortical activation

**Figure 4:**
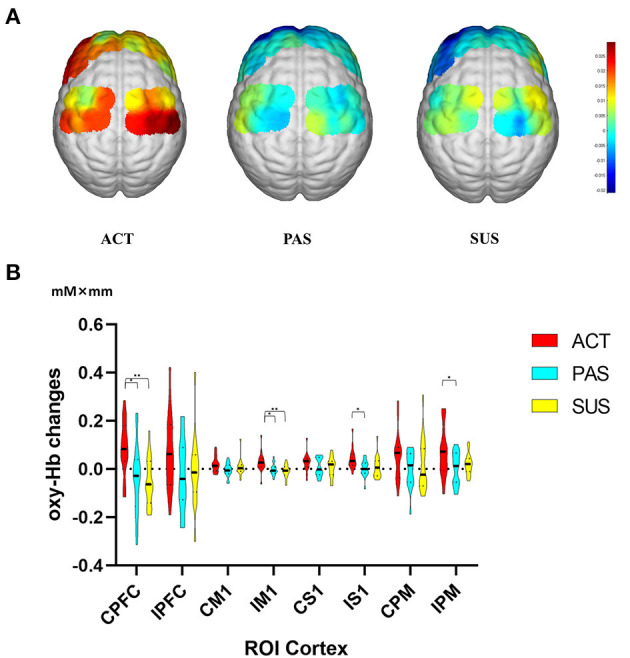
**(A)** Activation maps of the mean oxy-Hb changes under three different conditions. **(B)** Average changes in oxy-Hb concentration of ROIs for three conditions. Comparisons of execution-related oxy-Hb changes of ROIs for ACT, PAS, and SUS. C, contralesional; I, ipsilesional; oxy-Hb, oxy-hemoglobin; ROIs, regions of interest; PFC, prefrontal cortex; M1, primary motor cortex; S1, primary somatosensory cortex PM, premotor area, and supplementary motor cortex. *p < 0.05, **p < 0.01. Data are expressed as the mean with standard error (SE).

The activation maps of mean oxy-Hb changes under the three conditions are shown in the figure (Figure 4A).

One-way repeated measures ANOVA revealed significant differences in mean oxy-Hb changes among conditions in the three ROIs (Figure 4B): CPFC [F_(2, 48)_ = 6,798, *p* < 0.01], IM1 [F_(2, 48)_ = 6.733, *p* < 0.01], IS1 [F_(2, 48)_ = 4,392, *p* < 0.05], and IPM [F_(2, 48)_ = 3.658, *p* < 0.05]. Oxy-Hb responses in the CPFC region were stronger during ACT than during SUS (t = 3.363, *p* < 0.01) and PAS (t = 2.991, *p* < 0.05). Cortical activation in the IM1 was significantly greater during ACT than during SUS (t = 3.448, *p* < 0.01) and PAS (t = 2.812, *p* < 0.05). Oxy-Hb responses in the IS1 (t = 2.740, *p* < 0.05) and the IPM (t = 2.603, *p* < 0.05) were significantly higher during ACT than during PAS, and there is no significant difference in mean deoxy-Hb changes among conditions (data not shown).

**Figure 5:**
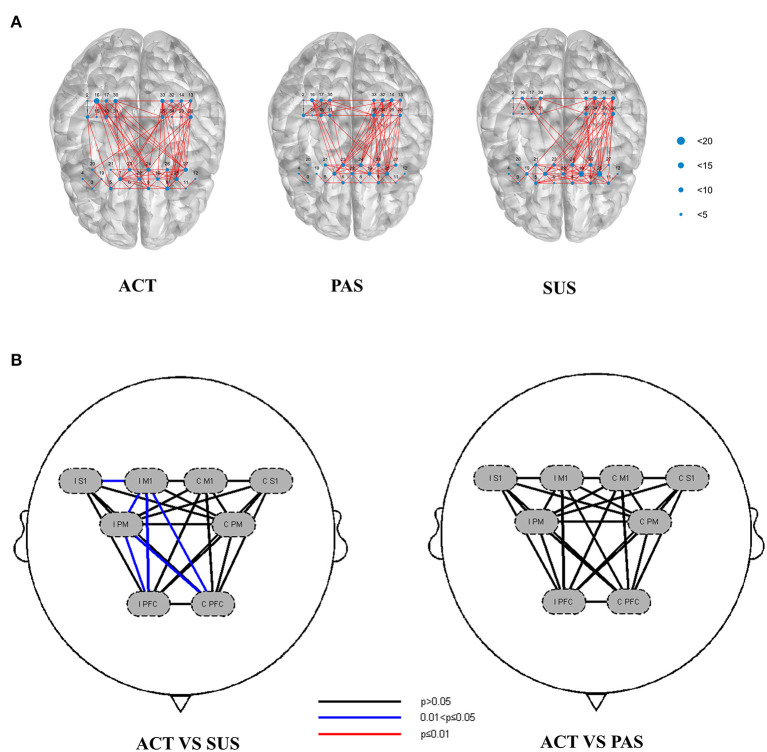
**(A)** Functional connectivity (FC) of ROIs during ACT, PAS, and SUS. **(B)** Channels with differences in FC between ACT and SUS (Pearson correlation), and there was no significant difference in FC between ACT and PAS. ROIs, regions of interest.

In the TS, the FC was analyzed by Pearson's correlation coefficient, and the FC of three types of training is shown in Figure 5A. Compared with during SUS, increased FC was observed during ACT, which is shown between the IM1 and ipsilateral S1 (t = 2.354, *p* < 0.05), the IM1 and IPM (t = 2.787, *p* < 0.05), the IM1 and IPFC (t = 2.581, *p* < 0.05), the IPM and IPFC (t = 2.880, *p* < 0.05), the IM1 and CPFC (t = 2.123, *p* < 0.05), the IPM and CPFC (t = 2.580, *p* < 0.05) (Figure 5B), and there was no significant difference in FC between the ACT and PAS measured.

## Discussion

The present study aimed to explore the activation mechanism and the FC of cerebral cortices in patients with stroke using fNIRS for upper limb intelligent feedback robot training.

The results showed that during ACT, higher cortical activation in the CPFC, IM1, and tighter FC between cortices was observed than during SUS, which supported our hypothesis. In the comparison between ACT and PAS, it found that the activation of cerebral cortices in the CPFC, IM1, and IPM of ACT was significantly stronger than that of PAS, but there was no significant difference in FC between the two groups, which was not consistent with our hypothesis. These findings are novel, as there are few studies on the activation of cerebral cortices in the process of upper limb robot training and traditional rehabilitation training, our research may reveal a part of why upper limb robot training is more effective than traditional rehabilitation training.

Both ACT and SUS were carried out by patients on their initiative, while SUS simply hung the upper limb of the affected side to allow patients to complete the movement repeatedly. In addition, ACT used an intelligent feedback robot, and patients played games in the face of a multimedia display screen in the process of training. Therefore, patients could be given visual, auditory, and other feedback to enable them to complete the whole training more actively. The fNIRS detection of the two types of training showed that hemodynamic responses in the CPFC and IM1 were higher during ACT than during SUS, which was consistent with previous studies. It has been reported that the real feedback group induced specific and concentrated brain activation in the left motor area after eight times of training, while the sham feedback group showed diffuse brain activation patterns throughout the cortex (31). Similarly, the literature has compared the real and sham feedback given to patients with stroke during upper limb functional training. After six times of training, the Fugl-Meyer (FM) of the real group was significantly better than that of the sham group, and the activation of the PM was enhanced (32). In addition, active cycling exercises with feedback increased the contralesional premotor cortex activation and improved cycling performance compared with those without feedback in patients with stroke (13). These findings suggest that providing immediate feedback during functional training in patients with stroke may enhance the activation of the cerebral cortices.

Compared with during SUS, increased FC was observed during ACT, which is shown between the IM1 and IS1, IM1 and IPM, IM1 and IPFC, IPM and IPFC, IM1 and CPFC, and IPM and CPFC. The reason for this result may be that patients were more motivated to achieve game goals during ACT. Moreover, the robot can better bear the weight of patients' upper limbs and adjust the friction of shoulder training, thereby reducing the participation of the contralesional cerebral cortex. This conjecture is similar to the principle of constraint-induced movement therapy. Movement of the affected limb helps patients to overcome the previously learned non-use for the affected limb and promotes use-dependent plastic brain reorganization. These two factors were regarded as the two therapeutic mechanisms of therapy (11). A study compared the effect of the affected upper limb side and bilateral exercise training in post-stroke patients. It found that compared with bilateral training, the coupling effect of CM1 to IM1 was significantly enhanced. In addition, the local efficiency of the IM1, IPFC, and CPFC and the hemispheric autonomy index of IPFC were significantly increased in unilateral training (12). It is proved that the stronger the motor motivation and the higher the participation of the affected limb, the stronger the FC of the brain on the ipsilesional side. Based on the principle of neuroplasticity of the brain, the reorganization of the brain structure and function occurs throughout the human lifespan (33). Meanwhile, a previous study has confirmed the positive correlation between functional improvement after stroke and enhanced neuroplasticity following rehabilitative interventions (34). In the process of nerve remodeling, if we carry out activation training on the ipsilesional side of the brain, restore the FC of the brain region, and reduce the compensatory activation of the contralesional side, the function may recover more quickly. A double-blind randomized controlled trial supports this conclusion. Mihara et al. performed six SMA facilitation with functional near-infrared spectroscopy-mediated neurofeedback treatments on patients with stroke. After treatment, the 3-meter Timed Up and Go (TUG) test of the intervention group was significantly better than that of the control group. Meanwhile, the intervention group showed significantly increased imagery-related supplementary motor area (SMA) activation and enhancement of resting-state connectivity between SMA and the ventrolateral premotor area (35). After Yuan trained stroke patients with robot-assisted upper limb training guided by the brain–computer interface 20 times, significantly modulated FC was observed between ipsilesional motor regions (M1 and SMA) and some contralesional areas (SMA and PM), and modulated FC with IM1 was significantly correlated with motor function improvement. In addition, increased interhemispheric FC among the SMC from resting-state data and increased LI from task-based data together indicated the re-balance of the two hemispheres during the recovery (36). This proved that the improvement of motor function and the functional changes between IM1 and the contralesional premotor area were significantly associated with the ipsilesional corticospinal tract integrity. Therefore, the more significant M1 activation and tighter FC of the ipsilesional brain during ACT may be the reasons why the curative effect is better than that of traditional rehabilitation therapy. We suspect that after two different treatments for a long time, the activation of the IM1 in the ACT group will be significantly higher than that in the SUS group, the FC of the ipsilesional brain will be tighter, and the function of the upper limb will be significantly improved. The next step of our research will verify this conjecture.

In the comparison between ACT and PAS, it found that the activation of the cerebral cortex in the CPFC, IM1, IS1, and IPM of ACT was significantly higher than that of PAS, but there was no significant difference in FC between the two groups, which was inconsistent with our hypothesis. The reason for this result may be the difference between active movement and passive movement. ACT requires patients to control muscle production for movement, in other words, it also requires more activation of the cerebral cortex, while PAS is passively activated by the robot. Therefore, the cortical activation of ACT is more significant. A previous study has compared the cortical activation of active left upper limb movement (on the affected sides in patients with stroke), with or without extrinsic motor performance visual feedback (LAV and LAnV), and passive left upper limb movement (affected sides in patients with stroke) (LP) in patients with stroke and healthy controls. The results showed that both the LAV and LAnV induced significantly higher activation in the ipsilesional SMA and the premotor cortex than in the LP, which indicates active upper-limb movement appears to induce higher cortical activation than that elicited by passive movement in both patients with stroke (37). However, there is no significant statistical difference in FC between the two groups. It may be that during PAS, patients received feedback from the outside world and carried out motor imagination, and the upper limbs were also passively completing the game goals, resulting in more FC between the brain regions. Studies have shown that both motor imagination and motor execution of finger activity in patients with stroke can lead to the reorganization of the brain network, and the same network dominated. In addition, M1 causes more exchange of causal information among motor areas during a motor execution task than during a motor imagery task due to its interaction with SMA, so the cortical activation of M1 will be stronger (38). Therefore, these findings suggest that PAS cannot significantly activate the cerebral cortices, but feedback stimulation and motor imagination during PAS can also activate brain regional connections and improve FC, which can be used in the treatment of patients with low myodynamia after stroke.

There are two limitations to this study. First, fNIRS detection is sensitive and easy to be affected by limb compensatory movement or the external environment. Although some improvements had been made in the training process, and motion artifacts were corrected using a technique based on moving standard deviation and spline interpolation, there are still different degrees of compensation and motion artifacts, which may interfere with the results. Follow-up research should improve the research design to overcome this problem. Second, the number of optodes used in this fNIRS study is not enough to cover the whole cerebral cortex, so we can only analyze the eight ROIs of bilateral PFC, PM, M1, and S1, but the activation of other cortices during training cannot be detected and cannot be analyzed completely. The follow-up study can use more channels of fNIRS instruments to detect more brain regions.

## Conclusion

In summary, this fNIRS study assessed the activation of cerebral cortices and the cerebral cortex network after stroke in different shoulder training processes and discussed the mechanism of upper limb intelligent feedback robot training. Our study shows that compared with SUS, ACT can activate cerebral cortices more strongly and show tighter brain connectivity on the ipsilesional side. This may be the reason why the curative effect of rehabilitation robot training is better than that of traditional rehabilitation training. In future research, we will compare the long-term effects of the two types of training to verify this conjecture. Compared with ACT, the cortical activation of PAS is weaker, but the FC of the brain regions has no statistical difference, which indicates that PAS can be used to treat patients with low myodynamia after stroke and enhance brain response. Our research is helpful to understand the difference in cerebral cortex activation between upper limb intelligent feedback robot rehabilitation and other types of rehabilitation training and provides a new method to explore the mechanism of brain response. It also provides an objective basis for the further application of upper limb intelligent feedback robots in the field of stroke rehabilitation.

## Data availability statement

The raw data supporting the conclusions of this article will be made available by the authors, without undue reservation.

## Ethics statement

The studies involving human participants were reviewed and approved by the Ethics Committee of Guangzhou Panyu Central Hospital. The patients/participants provided their written informed consent to participate in this study.

## Author contributions

XF and HL designed the experiment. LL and KG conducted the measurements and participated in the data acquisition. HG and WY prepared the figures and tables. XF supervised the whole process, data acquisition, analysis, manuscript revision, provided scientific input, and contributed to manuscript writing. HL and ZH participated in the manuscript revision, supervised the whole process, and provided clinical input. XF, HL, LL, KG, HG, ZH, and WY approved the final version of the manuscript. All authors contributed to the article and approved the submitted version.
